# Role of AI and Radiomic Markers in Early Diagnosis of Renal Cancer and Clinical Outcome Prediction: A Brief Review

**DOI:** 10.3390/cancers15102835

**Published:** 2023-05-19

**Authors:** Mohamed Shehata, Rasha T. Abouelkheir, Mallorie Gayhart, Eric Van Bogaert, Mohamed Abou El-Ghar, Amy C. Dwyer, Rosemary Ouseph, Jawad Yousaf, Mohammed Ghazal, Sohail Contractor, Ayman El-Baz

**Affiliations:** 1 Department of Bioengineering, University of Louisville, Louisville, KY 40292, USA; mnsheh01@louisville.edu; 2 Department of Radiology, Urology and Nephrology Center, Mansoura University, Mansoura 35516, Egypt; dr_rasha_taha@mans.edu.eg (R.T.A.); maboelghar@mans.edu.eg (M.A.E.-G.); 3 Department of Biology, Berea College, Berea, KY 40292, USA; gayhartm@berea.edu; 4 Department of Radiology, University of Louisville, Louisville, KY 40202, USA; eric.vanbogaert@louisville.edu (E.V.B.); sohail.contractor@louisville.edu (S.C.); 5 Kidney Disease Program, University of Louisville, Louisville, KY 40202, USA; amy.dwyer@louisville.edu (A.C.D.); rosemary.ouseph@louisville.edu (R.O.); 6 Electrical, Computer, and Biomedical Engineering Department, Abu Dhabi University, Abu Dhabi 59911, United Arab Emirates; jawad.yousaf@adu.ac.ae (J.Y.); mohammed.ghazal@adu.ac.ae (M.G.)

**Keywords:** renal cancer, artificial intelligence, radiomic markers, computer-aided diagnostic techniques, clinical outcome prediction

## Abstract

**Simple Summary:**

Renal cancer (RC) is ranked tenth among all types of cancer in men and women worldwide. Artificial intelligence (AI) and radiomics have allowed the development of AI-based computer-aided diagnostic/prediction (AI-based CAD/CAP) systems for noninvasive and precise diagnosis of RC and prediction of clinical outcome at an early stage. This, in turn, can conserve time, effort, and resources, ultimately benefiting both patients and healthcare providers. This review summarizes the studies from the last decade that used AI and radiomic markers for the early diagnosis of RC and prediction/assessment of clinical outcome/treatment response. Finally, a deep discussion, suggestions, and possible future avenues for improving diagnostic and treatment prediction performance is introduced, which might help fill the research gap.

**Abstract:**

Globally, renal cancer (RC) is the 10th most common cancer among men and women. The new era of artificial intelligence (AI) and radiomics have allowed the development of AI-based computer-aided diagnostic/prediction (AI-based CAD/CAP) systems, which have shown promise for the diagnosis of RC (i.e., subtyping, grading, and staging) and prediction of clinical outcomes at an early stage. This will absolutely help reduce diagnosis time, enhance diagnostic abilities, reduce invasiveness, and provide guidance for appropriate management procedures to avoid the burden of unresponsive treatment plans. This survey mainly has three primary aims. The first aim is to highlight the most recent technical diagnostic studies developed in the last decade, with their findings and limitations, that have taken the advantages of AI and radiomic markers derived from either computed tomography (CT) or magnetic resonance (MR) images to develop AI-based CAD systems for accurate diagnosis of renal tumors at an early stage. The second aim is to highlight the few studies that have utilized AI and radiomic markers, with their findings and limitations, to predict patients’ clinical outcome/treatment response, including possible recurrence after treatment, overall survival, and progression-free survival in patients with renal tumors. The promising findings of the aforementioned studies motivated us to highlight the optimal AI-based radiomic makers that are correlated with the diagnosis of renal tumors and prediction/assessment of patients’ clinical outcomes. Finally, we conclude with a discussion and possible future avenues for improving diagnostic and treatment prediction performance.

## 1. Introduction

Renal cancer (RC) is ranked tenth among all types of cancer in men and women worldwide. The number of renal cancer patients increases dramatically each year. In the USA, around 81,800 new cases of RC are expected to be diagnosed in 2023 [1,2], with approximately 14,890 patients expected to die [1,2]. Approximately, 67% of RC patients are diagnosed before developing metastasis and have a 5-year survival chance of 93%. The development of metastatic disease reduces the 5-year survival chance to 72% for local metastases and 15% for distant metastases, which becomes a serious life-threatening problem [1,2]. In 2022, the National Cancer Institute estimated an expenditure of USD 5.1 billion for RC care in the USA [3].

Renal cancer is a heterogeneous group of tumors which develop from different cell types within the kidney. Renal cell carcinoma (RCC) is considered the most common and aggressive type of RC, representing around 70% of all RC cases [4,5]. The clear-cell subtype of RCC (ccRCC) represents around 70% of RCCs, while non-clear-cell subtypes (nccRCC) make up the remaining proportion. These include papillary RCC (paRCC) and chromophobe RCC (chrRCC), which account for 15% and 5% of all RCCs, respectively [6]. According to the World Health Organization (WHO) [6], this taxonomy is of immense importance, as each RCC subtype has its own prognosis [6,7,8]. Using conventional diagnostic techniques, benign lesions such as angiomyolipoma (AML) and oncocytoma (ONC) can be easily misclassified as RCC [9,10,11,12,13], especially lipid-poor AML [14]. Misdiagnosis of such benign lesions can result in unnecessary surgical procedures. One estimate suggests that approximately 15–20% of tumors resected for a preoperative diagnosis of RCC might be AML [15]. Therefore, early and precise diagnosis of such renal tumors is critical to properly administer the optimal treatment plan.

Traditional methods to detect RC include complete blood count (CBC), in which red blood cells are counted; urine tests to check for blood, bacteria, or malignant cells; and blood tests which measure markers of renal function. Although these tests have the ability to suggest the presence of RC, they cannot provide an accurate diagnosis, subtype, grade, or stage. Biopsy remains the gold standard for a definitive diagnosis of RC [1,2]. However, it is not favorable due to its invasive nature, high cost, and relatively long recovery and diagnostic reporting time. Therefore, current research aims to find a reliable, cheap, fast, and noninvasive diagnostic technique which can accurately diagnose and precisely characterize RC at an early stage [16,17,18,19].

**Figure 1:**
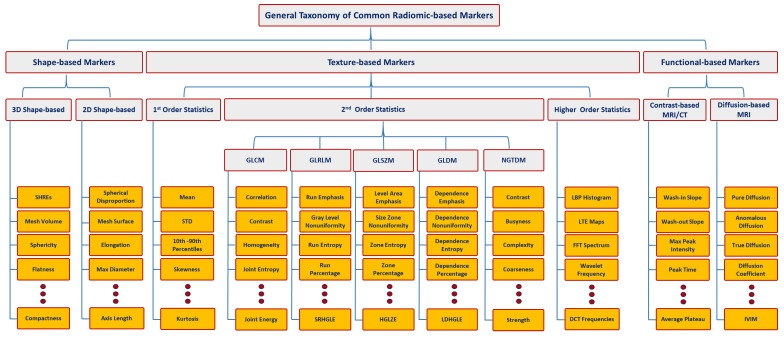
Taxonomy for different types of radiomic-based markers. Let MRI, CT, GLCM, GLDM, GLRLM, NGTDM, GLSZM, SHREs, STD, SRHGLE, HGLZE, LDHGLE, LBP, LTE, FFT, DCT, and IVIM denote magnetic resonance imaging, computed tomography, gray-level co-occurrence matrix, gray-level dependence matrix, gray-level run-length matrix, neighboring gray-tone difference matrix, gray-level size zone matrix, spherical harmonics reconstruction errors, standard deviation, short-run high gray-level emphasis, high gray-level-zone emphasis, large-dependence high gray-level emphasis, local binary pattern, Law’s texture energy, fast Fourier transform, discrete cosine transform, and intravoxel incoherent motion, respectively.

**Figure 2:**
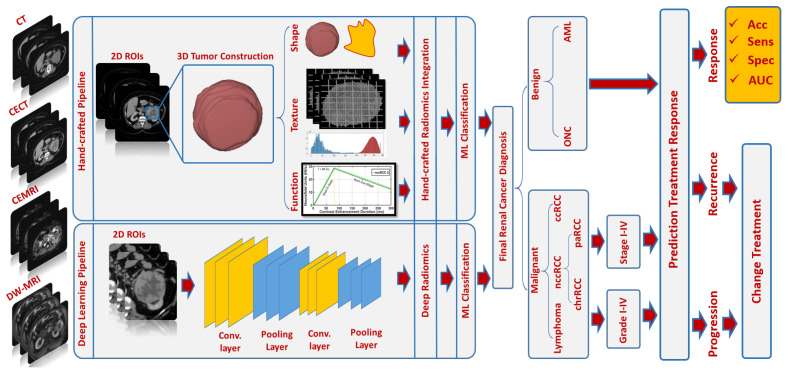
An illustrative example of an AI-based CAD/CAP pipeline for diagnosing renal tumors/prediction of treatment response using CT or MR images at an early stage.

Multiphasic (Phase 1: unenhanced or precontrast, Phase 2: arterial or corticomedullary, Phase 3: portal venous or nephrographic, and Phase 4: delayed or excretory) contrast-enhanced computed tomography (CECT) [20,21], multiphasic contrast-enhanced magnetic resonance imaging (CEMRI) [22], and diffusion-weighted MRI (DW-MRI) [23] are widely used for renal tumor diagnostic purposes. Radiomics techniques have been widely performed on CT and MR images to extract quantitative markers in different aspects, such as texture, morphology, and function, that characterize disease states [24,25] and could be used to improve diagnostic and prognostic accuracy for RC [26] at an early stage (see Figure 1). The new era and advances in the artificial intelligence (AI) field, including various machine learning (ML) and deep learning (DL) techniques, have demonstrated an important role, along with radiomics, in many clinical applications/practices. An illustrative example of an AI-based computer-aided diagnostic/prediction (AI-based CAD/CAP) pipeline to diagnose RC/predict treatment response is shown in Figure 2. Better diagnostic and predictive capabilities will allow for earlier intervention with an optimized management plan.

This survey reviews the studies from the previous decade that used AI and radiomic-based markers derived from CECT, CEMR, multiparametric MR, or DW-MR imaging modalities to produce AI-based CAD systems for diagnosing RC at an early stage. Specifically, the included studies aimed to identify a given renal tumor malignancy status [14,27,28,29,30,31,32,33], specify the associated subtype [22,33,34,35,36], and grade/stage the malignant tumors (I–IV) [22,37,38,39,40,41]. In addition to accurate diagnosis, treatment follow-up protocol is crucial to evaluate patients’ clinical outcome/treatment response, including the recurrence rate, overall survival (OS), and progression-free survival (PFS) rate. Therefore, we also review studies that were investigated in the same decade that use AI and/or radiomic markers to develop an AI-based and/or radiomic-based CAP system for prediction/assessment of treatment response for early stage tumors [42,43,44,45,46,47,48,49,50,51]. Lastly, we highlight the optimal radiomic markers that correlate with the diagnosis of renal tumors and prediction/assessment of clinical outcome/treatment responses that might help fill the research gap.

To identify such studies, we used different databases and search engines, including Google Scholar, PubMed, Web of Science, and ResearchGate. The following keywords were used individually or in combination, and our search was limited to the last decade: “Renal Tumors”, “Renal cancer”, “Renal Cell carcinoma”, “Clear Cell RCC”, “Non-Clear Cell RCC”, “Artificial Intelligence”, “Machine Learning”, “Computer-Aided Diagnosis”, “Diagnosis”, “Radiomics”, “Radiomic Markers”, “Texture Markers”, “Shape Markers”, “Functional Markers”, “Morphology”, “Histological Findings”, “Malignant”, “Benign”, “Angiomyolipoma”, “Oncocytoma”, “Chromophobe”, “Papillary”, “Subtyping”, “Grading”, “Staging”, “Computed Tomography”, “Magnetic Resonance Imaging”, “CT”, “CECT”, “MRI”, “CEMRI”, “DWMRI”, “Computer-Aided Prediction”, “Prediction”, “Treatment Response”, “Clinical Outcome”, “Recurrence”, “Overall Survival”, “Progression”, and “Therapy”. We found a total of 73 radiomic and AI-based studies, of which N = 64 concern the diagnosis of renal tumors and N = 9 concern the prediction of clinical outcome/treatment responses.

To the best of our knowledge, there is no agreement on the most reliable radiomic markers that can be used to develop a comprehensive AI-based system for the purposes of diagnosing renal tumors and predicting clinical outcome/treatment response simultaneously. After reviewing the state-of-the-art studies that have been developed in the last decade, we highlight the most common radiomic markers that can be correlated with both the diagnosis and treatment response prediction of renal tumors, potentially opening the door for future investigation and development of comprehensive diagnostic and predictive radiomic/AI-based systems.

## 2. AI-Based Diagnostic Studies

### 2.1. Computed Tomography (CT) Studies

In the differentiation of benign and malignant renal tumors, Hodgdon et al. and Yang et al. [14,27] discovered that first- and second-order texture markers of unenhanced CT (Phase 1) yielded an accuracy range of 82% to 91% and an area under the curve (AUC) range of 0.73 to 0.90 when using support vector machine (SVM) classifiers. A number of studies by You et al., Cui et al., Lee et al., and Feng et al. [28,52,53,54] found that first- and second-order texture markers of multiphasic CECT, along with SVM classifiers, achieved an accuracy range of 72% to 94% and an AUC range of 0.75 to 0.97. Yan et al. [55], Ma et al. [56], and Tang et al. [57] achieved comparable results with texture markers from multiphasic CECT. For instance, Yan et al. [55] employed artificial neural networks (ANNs) in conjunction with texture markers and attained 97% accuracy on a relatively small, unbalanced dataset (N = 50). Ma et al. [56] and Tang et al. [57] reported an AUC range of 0.67 to 0.93 using logistic regression (LR) classifiers in combination with texture markers. An expanded study by Ma et al. [56] found high accuracy using the nephrographic phase (Phase 3) of CECT with an AUC range of 0.74 to 0.89.

Nassiri et al. [58] extracted higher-order texture markers and shape markers from Phase 3 CECT and attained an accuracy range of 74% to 79% and an AUC range of 0.77 to 0.84 using Adaboost and random forests (RF) classifiers. Yap et al. [59] used the same markers extracted from multiphasic CECT and reported an AUC range of 0.65 to 0.75. Without the need for higher-order markers, Uhlig et al. [36] yielded an 84% accuracy and an AUC of 0.83 employing an RF classifier. Coy et al. [60] yielded the highest diagnostic accuracy of 74% using deep learning (DL) on Phase 4 (delayed phase) of CECT. Entropy as a first-order texture marker was extracted from unenhanced CT by Kim et al. [61] and was employed to distinguish between RCC and benign cysts using logistic regression (LR), achieving an AUC of 0.92.

Tanaka et al. [62] implemented a DL pipeline using the Inception-V3 convolutional neural network (CNN) and reported an accuracy range of 41% to 88% and an AUC range of 0.49 to 0.85, favoring Phase 2 CECT over other contrast phases. Li et al. [63] successfully distinguished benign ONC from malignant chrRCC by employing first- and second-order texture markers of multiphasic CECT along with an SVM classifier, resulting in 95% accuracy and an AUC of 0.85. The authors suggested that phases 2 and 3 outperformed other contrast phases for the specific task. In subsequent studies with larger datasets [64,65], they discovered that incorporating clinical factors improved the overall diagnostic accuracy. Meanwhile, Zabihollahy et al. [66] employed 2D and 3D CNNs in conjunction with semiautomated and automated tumor segmentation methods, reporting an accuracy range of 77% to 84%.

For RCC subtyping, Deng et al. and Yu et al. [34,67] demonstrated the efficacy of first-order texture markers, namely mean, standard deviation (STD), kurtosis, skewness, entropy, and median of Phase 3 CECT. Deng et al. [34] achieved 47% accuracy and an AUC between 0.80 and 0.84 using LR, while Yu et al. [67] yielded an AUC ranging from 0.86 to 0.92 using SVM. Furthermore, Shehata et al. [68] integrated shape, texture, and functional radiomic markers and obtained an accuracy between 79% and 98%, sensitivity from 0.89 to 0.95, and specificity from 0.91 to 1.00 using a multilayer perceptron artificial neural network (MLP-ANN). They also identified Phase 3 as the most useful phase for RCC subtyping. Zhang et al. [35] concurred with the significance of these markers but extracted them from Phase 2, achieving an accuracy of 78% to 87% and an AUC of 0.94 to 0.96 using an SVM classifier.

Verghase et al. [69] demonstrated that multiphasic CECT first-, second-, and higher-order texture markers are extremely important. They performed statistical analysis using stepwise LR and achieved an AUC range of 0.80 and 0.98. Uhlig et al. [36,70], in two subsequent studies, suggested first- and second-order texture markers, as well as shape markers obtained from Phase 3 CECT, demonstrating an accuracy range of 54% to 92% and an AUC of 0.45 to 0.85 using XGBoost and RF classification models. Finally, Chen et al. [71] promoted second-order texture markers of Phase 3 CECT and used LR to obtain an accuracy range of 82% to 88% and an AUC range of 0.86 to 0.90.

For RCC grading and staging purposes, Feng et al. [37] found that first-order texture markers such as entropy, STD, and kurtosis extracted from CECT are statistically significant. They reported an accuracy ranging from 70% to 79% and an AUC between 0.74 and 0.83. Shu et al. [38] found that first- and second-order texture markers, along with shape markers derived from CECT phases 2 and 3, could serve as valuable radiomic markers. Using the LR classification model, they yielded an accuracy of 72% to 78% and an AUC of 0.77 to 0.82. The same group extended their study by including a slightly larger cohort, excluding shape markers, and replacing the LR classifier with SVM and RF classifiers, resulting in an enhanced diagnostic accuracy of 92% to 94% and an AUC of 0.96 to 0.98. Two studies [72,73] focused on extracting second-order texture markers from CECT phases 2 and 3.

Ding et al. [72] achieved an AUC ≥ 0.67 after using LR classifiers, whereas Yin et al. [73] used SVM instead and yielded an enhanced AUC of 0.86. Second- and higher-order textures of Phase 3 CECT were reported particularly useful by Bektas et al. [74]. They relied on the evidence of achieving 85% accuracy and an AUC of 0.86 upon employing SVM classifiers. Lin et al. [75] determined that multiphasic CECT first- and second-order texture markers are useful in identifying renal tumors with 74% accuracy and an AUC of 0.87 using gradient boosting decision tree classifiers. Momenian et al. [76] posited that first-order texture markers in Phase 2 CECT have the potential to grade ccRCC tumors, achieving 97% accuracy using RF classifiers. Lai et al. [77] reported that first-order texture markers and shape markers of unenhanced CT can sufficiently classify ccRCC tumors using a Bagging classifier, resulting in an AUC of 0.75.

Luo et al. [78] achieved 81% accuracy and an AUC of 0.87 using RF classifiers on the derived first-order texture markers and shape markers from CECT phases 1 and 4, while first-, second-, and higher-order texture markers obtained from unenhanced CT were suggested by Yi et al. [79] to sufficiently grade ccRCCs, using an SVM classification model, with 90% accuracy and an AUC of 0.91. In line with Yi et al. [79], He et al. [80] agreed on the marker types, while disagreeing on the CECT phases from which they should be derived, instead recommending phases 2 and 3 of CECT and attaining an accuracy range of 91% to 94% using ANNs. Xu et al. [81] utilized an ensemble of various DL networks on 2D regions of interest (ROIs) of Phase 2 CECT and achieved 82% accuracy and an AUC of 0.88. Demirjian et al. [39] carried out a comprehensive investigation focused on both the grading and staging of ccRCC tumors. For grading, they employed multiple second-order texture markers in conjunction with the mean intensity, which served as a first-order texture marker, extracted from CECT. For the staging process, they relied exclusively on second-order texture markers. In both cases, they utilized RF classification models and attained AUC values of 0.73 and 0.77 for grading and staging, respectively.

Table 1 summarizes the above-mentioned AI-based CAD systems from the last decade that utilized CECT imaging in terms of the following attributes: study, main goal, data, radiomics, methods, results, and findings. Studies with the same main goal are grouped together for comparison purposes.

To sum up, the AI-based CAD systems that utilized CECT images demonstrated promising findings in the early diagnosis of RCC. These systems have effectively differentiated malignant from benign tumors with an accuracy range of 41% to 98% and an AUC range of 0.49 to 0.97, classified RCC tumor subtypes with an accuracy range of 47% to 92% and an AUC range of 0.49 to 0.92, and graded and staged RCC tumors with an accuracy range of 70% to 97% and an AUC range of 0.67 to 0.98. Entropy, a first-order texture marker, has frequently been identified as a crucial radiomic marker extractable from multiphasic CECT. Phases 2 and 3, namely the arterial phase/corticomedullary phase and portal venous/nephrographic phase, have been the most commonly used and recommended. Furthermore, machine learning classifiers such as LR, RF, SVM, and ANN have yielded the best classification results. While CECT has proven sufficient in RCC diagnosis, it is not the preferred modality when radiation exposure is contraindicated (e.g., in pregnant or pediatric patients). This has prompted researchers to explore the capabilities of alternative imaging modalities, such as MRI, to avoid radiation exposure whenever possible. Our search within the last decade revealed a limited number of studies on this topic, which we discuss in detail below.

### 2.2. Magnetic Resonance Imaging (MRI) Studies

In the differentiation of benign and malignant renal tumors, Xu et al. [29] investigated the potential of DL and ML using T2-weighted MRI and DW-MRI. Their study included a total of 217 patients with renal tumors, allocating 173 patients to the training set and 44 patients to the testing set. Following manual identification of ROIs, the investigators used three distinct DL ResNet-18 models and three separate handcrafted-based RF models, incorporating a total of 96 radiomic markers. The first model used T2-weighted imaging, the second model used DW-MRI, and the third model combined both modalities. The ResNet-18 models demonstrated accuracies of 77%, 80%, and 81.3%, while the handcrafted RF models attained accuracies of 77%, 71%, and 82%. Oostenburgge et al. [30] conducted a study to evaluate texture markers derived from 3D ADC maps of DW-MR images for distinguishing benign ONC from malignant RCC. The dataset comprised 39 renal tumors, including 32 RCCs and 7 ONCs. The authors found that entropy, STD, tumor volume, and gender demonstrated statistical significance among the different tumor groups. By integrating these markers, they achieved an AUC of 0.91 with 86% sensitivity and 84% specificity using the LR classification model. Furthermore, they discovered that entropy and the 25th percentile were statistically significant when comparing healthy cortical regions with tumor tissue.

Li et al. [31] included 92 DW-MRI renal tumors, with malignant tumors including (ccRCC, N = 38), (paRCC, N = 16), and (chrRCC, N = 18) and benign tumors comprising (AML, N = 13) and (ONC, N = 7). The authors constructed 3D ADC maps and calculated 10 distinct first-order texture markers. Following statistical analysis to identify significant markers, they evaluated diagnostic performance using ROC analysis. They reported that the mean, median, 75th percentile, 90th percentile, STD, and ADC entropy of malignant tumors were significantly higher than those of benign tumors. They reported 80% sensitivity, 86.1% specificity, and an AUC of 0.85%. Razik et al. [23] investigated multiparametric MRIs of 54 renal masses, including (RCC, N = 34), (AML, N = 14), and (ONC, N = 6), obtained from 42 patients. The researchers placed 2D ROIs on the maximum area of each tumor and extracted six first-order texture markers. Through ROC analysis, the mean of positive pixels (MPP) demonstrated the best diagnostic performance in separating RCC from AML (AUC = 0.89) on b_500_ s/mm^2^ of DW-MRI. Furthermore, the mean value was identified as the best marker for distinguishing between RCC and ONC (AUC = 0.94) on b_1000_ s/mm^2^ of DW-MRI.

Nikpanah et al. [92] explored the potential of deep CNNs along with T2-weighted MRI and multiphasic CEMRI for differentiating ccRCC from ONC. Their study included 74 patients with a total of 243 renal masses, comprising 203 ccRCC and 40 ONC tumors. The researchers placed 2D ROIs around the tumors and input them into an AlexNet CNN model, achieving 91% accuracy and an AUC of 0.9. Arita et al. [93] analyzed the texture of 3D ADC maps derived from DW-MRI to distinguish between benign AML and malignant nccRCC. They encompassed a training dataset of 67 tumors (AML = 46 and nccRCC = 21) and a validation dataset of 39 tumors (AML = 24 and nccRCC = 15). A total of 45 texture markers were extracted, and the long-zone high gray-level emphasis, as a second-order texture marker, was reported as the most dominant marker for identifying AML. Their RF classification model yielded an AUC of 0.82, which was comparable to the radiologic assessment.

Gunduz et al. [94] used texture analysis of ADC maps for distinguishing benign ONC from malignant chrRCC in a small cohort of 14 patients (ONC = 6 and chrRCC = 8). The study identified six texture markers, with five being second-order (run variance, short-run emphasis, normalized run-length nonuniformity, run percentage, long-run emphasis) and one being first-order (square root of mean ADC). They achieved 87.5% sensitivity and 83% specificity using ROC analysis. Matsumoto et al. [32] explored texture analysis on DW-MRI for differentiating between AMLs and ccRCCs. Their study consisted of two datasets. The first dataset comprised 83 tumors (AML = 18 and ccRCC = 65) that were used for the development of the diagnostic model, while, the second dataset included 39 tumors (AML = 13 and ccRCC = 17), serving as external validation. From the ADC maps, they extracted 39 texture markers and employed an RF model to determine the importance of these markers. They identified the mean ADC value as a significant first-order texture marker and both long-run low gray-level enhancement and gray-level run emphasis as dominant second-order texture markers in the diagnostic process, achieving an AUC of 0.87.

For RCC subtyping and grading, Goyal et al. [22] examined the power of texture markers derived from multiparametric MRI techniques, including T1-weighted MRI, T2-weighted MRI, multiphasic CEMRI, and DW-MRI. Using a total of 34 renal masses, consisting of 29 ccRCCs (low-grade = 19, high-grade = 10) and 5 nccRCCs, they placed 2D ROIs on the maximum viable tumor area. Then, they extracted multiple first-order texture markers, including mean, entropy, STD, skewness, MPP, and kurtosis, from each MRI sequence for further investigation. For RCC subtyping, using ROC analysis, entropy attained an AUC of 0.81 on T2-weighted MRI, STD yielded AUCs of 0.81 and 0.88 on DW-MRI at b_500_ and b_1000_ s/mm^2^, respectively, mean yielded an AUC of 0.848 on ADC maps, and skewness reached an AUC of 0.85 on T1-weighted MRI and 0.91 on Phase 2 CEMRI. In the grading of ccRCC tumors, entropy yielded an AUC of 0.82 on DW-MRI at b_1000_ s/mm^2^, mean attained an AUC of 0.89 on Phase 2 CEMRI, and MPP achieved an AUC of 0.87 on Phase 3 CEMRI. The authors suggested that various first-order texture markers derived from multiparametric MRIs could serve as valuable diagnostic tools for both subtyping and grading renal tumors. Sun et al. [40] explored the power of texture analysis on susceptibility-weighted magnetic resonance imaging (SW-MRI) to grade ccRCCs. The study encompassed 45 patients, comprising 29 low-grade and 16 high-grade ccRCC tumors. The total number of derived texture markers was reduced from 396 to 10. Using multivariable logistic regression, the authors constructed a diagnostic model which produced 77.3% accuracy, 80.5% sensitivity, and 71.4% specificity.

Chen et al. [41] aimed at grading ccRCC using Phase 2 CEMRI. Their study included 99 tumors, with 61 low-grade and 38 high-grade cases. They placed 2D ROIs, then extracted and analyzed various first-, second-, and higher-order texture markers. Using RF importance analysis, six texture markers, namely entropy, sum of entropy, kurtosis, horizontal gray-level nonuniformity, gray-level nonuniformity, and run-length nonuniformity, were selected. Subsequently, they achieved 86.2% accuracy, 72.7% sensitivity, 94.4% specificity, and an AUC of 0.76 on the validation dataset (N = 29) using a modeled MLP-ANN classifier. Despite high specificity, the sensitivity was relatively low, which could be attributed to class imbalance. The utility of various radiomic markers, including shape markers and first- and second-order texture markers, extracted from T2-weighted and multiphasic CEMRI was explored by Choi et al. [95] to grade ccRCC. Their study encompassed 364 renal tumors, of which 272 were low-grade and 92 were high-grade. Their RF classification model demonstrated 98% accuracy, 72% sensitivity, 95% specificity, and an AUC of 0.89. Although the overall diagnostic performance was satisfactory, the relatively low sensitivity was likely attributable to data imbalance.

Hoang et al. [96] explored diagnosing renal RCC using texture analysis of multiphasic MRI. Their study involved 212 renal lesions, of which 96 were normal, 11 were ONC, 87 were cRCC, and 8 were paRCC. These lesions were divided into two halves for training and validation purposes. Following the placement of 2D ROIs, first-order texture markers, including mean, skewness, STD, and kurtosis, were extracted. Using an RF classifier among all phases, Phase 1 CEMRI demonstrated the best diagnostic accuracy of 79.1%. However, integrating texture markers from different phases raised the final diagnostic accuracy to 83.7%. In a following study by the same researchers [33], the utility of multiphasic CEMRI for differentiating benign from malignant renal tumors and distinguishing major subtypes of RCCs was investigated. The study included 140 renal lesions, of which 30 were ONC, 90 were RCC, and 22 were paRCC. After placing 2D ROIs on the slices encompassing the largest cross-section in each contrast phase, multiple first- and second-order texture markers were extracted using histogram analysis, gray-level co-occurrence matrix (GLCM), gray-level run-length matrix (GLRLM), gray-level size-zone matrix (GLSZM), and neighborhood gray-tone difference matrix (NGTDM). Least absolute shrinkage and selection operator (LASSO) regression was then applied to select the optimal markers for classification. The study concluded that first-order texture markers were useful to identify malignancy, while adding second-order texture markers improved the accuracy of subtyping. In terms of classification, they used RF classification models and reported 77.9% accuracy in distinguishing between paRCC and ccRCC, 79.3% accuracy in differentiating between ONC and ccRCC, and 77.9% accuracy in discriminating between ONC and paRCC.

Table 2 summarizes the above-mentioned AI-based CAD systems from the last decade that utilized different MRI modalities in terms of the following attributes: study, main goal, data, radiomics, methods, results, and findings. Studies with the same main goal are grouped together for comparison purposes.

To sum up, the AI-based CAD systems that utilized various types of MRIs demonstrated interesting results and findings in the early diagnosis of RCC. These systems achieved an accuracy range of 77% to 91% and an AUC range of 0.82 to 0.91 for differentiating malignant from benign tumors. Furthermore, they attained an accuracy range of 77% to 98% and an AUC range of 0.76 to 0.89 for subtyping and/or grading RCC tumors. First-order texture markers such as entropy, MPP, mean, skewness, and kurtosis have been frequently identified as the most dominant and important radiomic markers derived from multiparametric MRIs. These markers are useful for differentiating between benign and malignant renal tumors. The addition of second-order texture markers derived from GLRLM has also proven valuable. Notably, texture analysis of ADCs derived from DW-MRI was the most commonly used technique among the reviewed MRI studies. Additionally, RF classifiers were chosen by the majority of these studies, yielding superior classification results. In spite of MRIs being useful for identifying malignancy status, subtyping RCCs, and grading RCCs, there is a lack of research investigating the staging of RCCs. Staging is crucial for determining a tumor’s spread, size, and location, making it a vital area for future investigation.

## 3. AI-Based Prediction of Clinical Outcome/Treatment Response Studies

A limited number of studies have explored the role of AI and/or radiomics derived from CT and/or MRI in predicting clinical outcomes and treatment responses in RCC patients. Focusing on MRI studies, Bharwani et al. [43] investigated the potential correlation between various radiomic markers extracted from DW-MRI and dynamic contrast-enhanced MRI (DCE-MRI) and the response to neoadjuvant sunitinib therapy, specifically overall survival (OS), in metastatic RCC (mRCC). Their study included 20 mRCC patients who survived after completing three treatment cycles. By placing 3D ROIs on DW-MR images, they calculated tumor volume, constructed ADC maps, and generated ADC histograms. They then determined mean ADC, AUC_low_ (ADC 25th percentile), kurtosis, and skewness before and after treatment. Using 2D ROIs on DCE-MR images, they computed the maximum signal intensity and the wash-in rate before and after treatment. Using the Kaplan–Meier (KM) method, they analyzed OS by dividing mRCC patients into two groups based on the median of the aforementioned descriptive statistics as a cutoff. Their findings revealed that patients with a tumor volume below the median at baseline experienced a prolonged OS. An increase in AUC_low_ of ADCs greater than the median was indicative of reduced OS, whereas a decrease in AUC_low_ suggested a prolonged OS. Moreover, a positive correlation was found between mean ADC in the primary tumor and metastases.

Antunes et al. [44] conducted a study aiming to identify the optimal radiomic markers on an integrated positron emission tomography (PET)/MRI that best describe early treatment responses and changes in advanced mRCC patients (N = 2) undergoing sunitinib therapy. They extracted a total of 66 radiomic markers, including raw T2w signal, postprocessed T2w, 30 postprocessed T2w textures, raw ADC map, 30 ADC textures, standard uptake value (SUV), and 2 PET textures. Subsequently, they employed a scoring function to determine the top 25 ranked radiomic markers in the two patients under study. They found that SUV from PET, T2w difference average from T2w, and ADC energy from DW-MRI ADC maps were ranked the highest among the 25 radiomic markers in terms of reproducibility and capturing treatment-related changes or responses. Furthermore, the integration of these radiomic markers resulted in improved prediction performance. However, they acknowledged that their findings are limited due to a small sample size of only two patients and emphasized the need for further investigation in a larger cohort to validate their results.

Reynolds et al. [51] conducted a study to investigate the potential of radiomic markers derived from DW-MRI (N = 12) and DCE-MRI (N = 10) as predictors of early treatment response in RCC patients following stereotactic ablative body radiotherapy (SABR). For shape markers, 3D ROIs from CT images were utilized to contour the tumor, and tumor volume was calculated at baseline and after three different follow-up scans to estimate tumor volume change. For textural markers, ADC maps were derived from 3D ROIs of DW-MR images, and an ADC histogram was constructed. Mean ADC, median, kurtosis, and skewness were subsequently calculated. Employing DCE-MR images, various functional markers were estimated, including mean T_onset_, mean IRE, mean MaxE, mean T_wout,_ mean IRW, mean K_trans_, iAUCAC60, % washout voxels, % plateau voxels, % persistent voxels, and % nonenhancing voxels. Spearman rank correlation coefficients (ρ) were computed to compare changes in the aforementioned parameters against the % change in tumor volume. Statistically significant correlations were observed between the change in percentage washout, change in mean IRE, and mean K_trans_ and the change in tumor volume (p<0.05). Changes in ADC kurtosis also demonstrated statistically significant positive correlations with % tumor volume change (p<0.05).

For CT studies, Lubner et al. [50] conducted a study to identify radiomic texture markers that can be extracted from phases 1 and 3 of CECT images in RCC patients (N = 157) and might be correlated with histological findings and treatment response. From 2D ROIs, and after applying various texture filters, they extracted six different first-order texture markers: mean, STD, MPP, entropy, skewness, and kurtosis. Their study found that entropy, STD, and MPP were correlated with histologic type, nuclear grade, and clinical outcomes (time to recurrence and OS) in patients with RCC.

Boos et al. [45] assessed the ability of mean and median intensity attenuation, represented by Hounsfield units (HU) and estimated from CECT images, in predicting treatment responses (response, stable, and progression) in patients with RCC tumors (N = 19) who received targeted therapy, specifically vascular endothelial growth factor receptor (VEGFR) tyrosine kinase inhibitors (TKI). After estimating the mean and median HUs, they performed the Wilcoxon signed-rank test to compare the change between the baseline and consecutive post-treatment scans for overall outcome assessment. They concluded that the median HU attenuation shift provided better prediction accuracy (79%) than the mean (74%) and thus is preferable. Moreover, a shift in median <–44 HU indicated a partial response, while a shift in median >–41 HU indicated progression, and therefore, median HU shift correlates well with clinical outcomes in mRCC patients.

Haider et al. [46] conducted a study to highlight potential radiomic predictors of PFS and OS that could be extracted from CECT images in RCC patients (N = 40) undergoing treatment with sunitinib. After placing 2D ROIs on renal tumors, they extracted various first-order texture markers, such as MPP, STD, skewness, kurtosis, and size-normalized STD (nSTD), and entropy as a second-order texture marker. A Cox proportional hazards survival statistical analysis was employed to determine the predictors of both PFS and OS. Their study revealed that nSTD extracted at baseline and after treatment is positively correlated with both OS and PFS, while entropy and tumor size changes are predictors of OS but not PFS.

Mains et al. [47] investigated radiomic functional markers derived from CECT images that could potentially predict OS and PFS in mRCC patients (N = 69). After placing 2D ROIs, they identified seven markers that describe functionality, specifically, blood volume using deconvolution (BV_deconv_), blood flow using deconvolution (BF_deconv_), standardized perfusion values using deconvolution (SPV_deconv_), blood volume using maximum slope (BV_max_), standardized perfusion values maximum slope (SPV_deconv_), blood volume using the Patlak model (BV_patlak_), and permeability surface area product using the Patlak model (PS). They applied various statistical analysis methods on the histogram data of the aforementioned markers and found that medians and modes of BV_deconv_, BV_patlak_, and BF_deconv_ are statistically significant (*p* < 0.05) and have the strongest correlation with clinical outcomes (PFS and OS).

Khodabakhshi et al. [48] conducted a study to investigate possible radiomic markers extracted from Phase 2 CECT along with clinical biomarkers for the prediction of OS in RCC patients after partial or radical nephrectomy (N = 210). The 2D ROIs were manually drawn, and a total of 225 radiomic markers were extracted, including 29 shape markers, 50 first-order texture markers, and 136 second-order texture markers. Additionally, 59 clinical markers were included in the analysis. They employed Cox proportional hazards regression as a marker selection method, which resulted in a reduced set of 11 radiomic markers and 12 clinical markers. Then, they applied the accelerated failure time technique to specify the contribution of the selected markers on OS time. Their study revealed that flatness, area density, and median were the most significant radiomic markers (*p* < 0.05), while tumor heterogeneity, grade, and stage were the most significant clinical markers (*p* < 0.05). Therefore, all of these markers combined were significant predictors for OS in RCC patients.

Zhang et al. [49] investigated the prediction potential of radiomic markers extracted from CECT images and clinical markers linked to PFS after partial or radical nephrectomy in ccRCC (N = 175). After manual segmentation of tumors using 3D ROIs, they extracted a total of 428 radiomic markers (107 per CT phase). They then applied the least absolute shrinkage and selection operator with 5-fold cross-validation (LassoCV) to select the dominant markers, resulting in six markers (four shape-based markers and two second-order texture markers) as follows: least axis length (Phase 2), maximum 2D diameter row (Phase 4), surface volume ratio (Phase 1), maximum 2D diameter slice (Phase 3), size-zone nonuniformity (Phase 2), and complexity (Phase 2). Subsequently, they established a multivariate Cox regression model using a training dataset (N = 125) for PFS prediction and saved the other 50 subjects for validation. This model depended on a weighted sum of the selected markers. In addition, they integrated statistically significant clinical markers (age, clinical stage, and Karnofsky performance status (KPS) score), resulting in a PFS prediction model encompassing both clinical and radiomic markers. After validating their model on the validation dataset (N = 50), they achieved an accuracy of 70%, sensitivity of 58%, specificity of 74%, and an AUC of 0.71. They concluded that radiomic-based markers extracted from CECT, especially Phase 2, demonstrated better prediction performance of PFS in ccRCC patients when combined with clinical markers.

Table 3 provides a summary of the aforementioned radiomic-based CAP systems developed in the last decade using different CT and/or MRI modalities. The Table includes the following details: study, main goal, radiomics, methods, results, and findings.

In summary, these few studies investigated the potential of developing radiomics-based CAP systems utilizing various types of CT and MRI scans, showing promising results in early prediction of treatment response, including overall survival rate, progression-free survival, and time to recurrence. Most of these studies relied on statistical analyses to identify statistically significant radiomic markers correlated with specific clinical outcomes or treatment responses. Histogram measures of ADC maps extracted from DW-MR images, particularly changes in mean ADC, ADC energy, and ADC kurtosis, were significant predictors of clinical outcomes (p<0.05) [43,44,51]. Additionally, changes in radiomic-based functional parameters extracted from DCE-MR images, namely changes in percentage washout, mean IRE, and mean K_trans_, demonstrated significant correlations with changes in tumor volume (p<0.05) and thus are potential indicators for clinical outcomes or treatment responses [51]. First-order texture markers, specifically entropy, STD, and MPP, are correlated with time to recurrence and OS [46,50], while nSTD is positively correlated with both OS and PFS [46]. Functional-based radiomic markers, such as median HU, are potential predictors of partial response and progression [45], while medians and modes of BV_deconv_, BV_patlak_, and BF_deconv_ are strong predictors of OS and PFS. Moreover, shape-based radiomic markers, namely flatness and area density, extracted from Phase 2 CECT are strong predictors of OS [48], while least axis length extracted from the same phase of CECT is a potential indicator of PFS [49]. Some clinical markers can be combined with radiomic markers for enhanced prediction performance of PFS, such as age, stage, and KPS score [49], and for improved prediction of OS, such as grade and stage [48]. Despite these promising findings, there is considerable heterogeneity and diversity in the number of patients included in each study, the type of treatment administered before or after nephrectomy (e.g., radiation therapy, targeted therapy, neoadjuvant therapy, etc.), and the final goals or endpoints of these studies (e.g., type of clinical outcome). Furthermore, most of these studies were primarily statistical-based in nature and were not aimed at the development of a comprehensive automated AI-based CAP system.

## 4. Discussion and Future Directions

The success of accurate and timely diagnosis of renal tumor malignancy status, specific subtype, and associated grade (I–IV) and stage (I–IV) holds significant clinical importance, as it directly influences the determination of appropriate treatment and management plans. As a result, precise prediction of clinical outcomes or treatment responses, including recurrence rate, overall survival rate, and progression-free survival rate, is essential to avoid the burden of unnecessary treatment strategies. This, in turn, can conserve time, effort, and resources, ultimately benefiting both patients and healthcare providers.

**Suggested Diagnostic Radiomic Markers:**
  - In terms of differentiating malignant from benign renal tumors, CT studies have demonstrated a slightly higher diagnostic accuracy [52,54,55,63,68,83] when compared with the results obtained by MRI studies [29,31,92,93]. This can be partially attributed to the superior resolution provided by CT in comparison with MRI. In both imaging modalities, first-order texture markers, including entropy, mean, MPP, skewness, and kurtosis, were reported to be sufficient for the intended purpose.
  - For subtyping and grading, both CT [68,71,73,74,76,79,80,88,89,90,91] and MRI [22,41,95,96] studies exhibited adequate diagnostic performance, suggesting that second-order texture markers, particularly those derived from the GLCM and GLRLM, should be combined with first-order texture markers. A limited number of studies have relied on morphological or functional markers, which, if integrated, could significantly enhance diagnostic performance [68]. In this context, both imaging modalities can be utilized for subtyping and grading purposes. However, MRIs are preferable in cases involving pediatric patients or pregnant women [97] to prevent exposure to ionizing radiation. For staging, a few CT studies demonstrated promising diagnostic performance [39], while MRI studies did not investigate radiological staging.

**Suggested Diagnostic Radiomic Techniques:** Generally, handcrafted radiomic techniques were more commonly investigated in both CT [14,27,28,34,35,37,54,62,68,74,82,86] and MRI [22,23,29,30,32,93,94] studies, as opposed to deep learning radiomic techniques, which were less frequently utilized in CT [66,81,83,88,95] and MRI [92] studies. Handcrafted techniques have proven efficient, as evidenced by high diagnostic accuracy, sensitivity, and specificity, as well as being well-understood (i.e., explainable AI), making them desirable and dependable.
**Suggested Diagnostic Classifiers:** The RF, SVM, and ANN classifiers were the most frequently utilized AI-based classification models in CT studies [14,27,28,35,36,38,39,52,53,54,55,58,59,67,68,70,74,76,78,79,80,85,86,89,90], while the RF classifier was predominantly selected in MRI studies [29,32,33,41,93,95,96]. These classifiers have provided impressive diagnostic results and have been widely accepted by researchers in the field due to their ability to handle nonlinear and multiclass classification problems.
**Suggested Imaging Modalities/Phases:** Contrast-enhanced phases 2 and 3 (corticomedullary/, arterial phase, and nephrographic/portal venous phase) were reported to be the most informative phases for extracting radiomic markers in both CT [35,36,38,58,62,64,68,70,71,72,73,74,75,76,80,81,87,89,90,91] and MRI [41] studies. Meanwhile, texture analysis of ADCs on DW-MRI was the most commonly employed technique to extract radiomic markers in MRI studies [29,30,31,32,94].
**Suggested Prediction Radiomic Markers:** In terms of treatment response prediction, entropy, mean, skewness, kurtosis, STD, and median have been identified by most CT studies [46,50] as potential radiomic markers for predicting OS and PFS. On the other hand, histogram measures of ADC maps extracted from DW-MR images, specifically changes in mean ADC, ADC energy, and ADC kurtosis, were the most promising predictors of clinical outcome in MRI studies [43,44,51]. To the best of our knowledge, no studies have employed AI, ML, or DL for the purpose of predicting treatment response; rather, they have relied on statistical analyses to identify significant markers correlated with clinical outcome/treatment response. A limited number of studies have depended on morphological or functional markers, which, if integrated, could significantly enhance clinical outcome/treatment response prediction [43,46,48,49,51].

It is worth noting that one study [50] attempted to identify optimal radiomic-based markers correlated with both histological findings and treatment responses. The authors concluded that first-order texture markers, specifically entropy, STD, and MPP extracted from phases 1 and 3 of CECT, were correlated with histological type, nuclear grade, and clinical outcomes (time to recurrence and OS) in patients with RCC. However, this study did not attempt to incorporate these radiomic markers into a comprehensive AI-based CAD/CAP system capable of simultaneously diagnosing RC and predicting treatment response, which is the ultimate goal of this survey. Consequently, the research gap remains and warrants further investigation.

**Future Directions:** While renal cancer diagnosis is a well-established research area, with numerous CT and MRI studies having developed radiomic and AI-based CAD systems for determining malignancy status, subtyping, grading, and staging, some investigations still suffer from low sensitivity or specificity [36,38,40,53,58,60,66,78,82,87,89,93,96]. Consequently, integrating radiomic markers extracted from multiple imaging modalities, such as CT and MRI, may improve diagnostic performance. Furthermore, as radiological-based analysis may not be sufficient for predicting clinical outcome/treatment responses, incorporating histopathological image analysis that captures characteristics such as cell color, shape, size, and staining could enhance prediction capabilities. Identifying robust AI models may reduce subjectivity by pinpointing optimal markers for treatment response prediction purposes. It is worth noting that a new trend in predicting treatment response using radiogenomics has recently emerged in a few studies and requires further investigation [98,99,100,101,102,103].

In conclusion, more investigative studies are still ongoing for both CT and MRI for the purpose of diagnosing renal tumors, as well as predicting clinical outcome/treatment responses for optimal management plans. Progress in the early diagnosis of renal tumors and treatment response prediction depends mainly on the identification of optimal discriminating markers for the intended diagnostic/prediction problems, as well as the development of robust, reproducible, and generalizable AI-based diagnosis/prediction models. By providing these future directions and suggestions, we aim to encourage investigators and researchers to address this research gap and achieve the intended goal of establishing a comprehensive, unified CAD/CAP system that can be reliably used for both renal tumor diagnosis and clinical outcome/treatment response prediction, ultimately leading to improved healthcare outcomes.

## Figures and Tables

**Table 1:** Summary of the last decade’s CT-based studies on early diagnosis of renal tumors.

Study	Data	Radiomics	Methods	Results	Findings
**Main Goal(s): Benign vs. Malignant**					
Yang et al. [27]	AMLwvf vs. RCC (N = 163) Multiphasic CECT	Shape: 12 1st-Order Statistics: 17 2nd-Order Statistics: 74 – GLCM: 23 – GLRLM: 16 – GLSZM: 16 – NGTDM: 5 – GLDM: 14	ROI: 2D Radiomics: Pyradiomics Classification: SVM, 5-fold CV	Acc: 0.82 Sen: 0.83 Spe: 0.78 AUC: 0.90	Radiomics extracted from unenhanced CT phase can be used to precisely discriminate AMLwvf from RCC using SVM
You et al. [28]	AMLwvf vs. RCC (N = 67) Multiphasic CECT	1st-Order Statistics: 3 – Phase 1: 2 – Phase 4: 1 2nd-Order Statistics: 2 – Phase 2: 1 (GLCM) – Phase 3: 1 (GLRLM)	ROI: 2D Radiomics: In-house software, SFS Classification: SVM, k-fold CV	Acc: 0.85 Sen: 0.82 Spe: 0.76 AUC: 0.85	Radiomics of small renal masses extracted from multiphasic CECT can accurately differentiate between AMLwvf and ccRCC using SVM
Coy et al. [60]	AMLwvf vs. RCC (N = 179) Multiphasic CECT	RGB encoding of the whole tumor volume in Phase 4	ROI: 3D Radiomics: TL of GTf Classification: TL of GTf, k-fold CV	Acc: 0.74 Sen: 0.86 Spe: 0.44 AUC: —	Radiomics extracted from 3D VOI of the entire tumor shown a reasonable diagnostic accuracy using Phase 4 CECT and TL of GTf
Deng et al. [82] (Study 1)	Benign vs. RCC (N = 501) Phase 3 CECT	1st-Order Statistics: 5 – entropy – kurtosis – skewness – mean – max	ROI: 2D Radiomics: TexRAD software, LSSF Classification: Binary LR (Statistical analysis only)	Acc: 0.47 Sen: 0.31 Spe: 0.86 AUC: 0.62	Entropy had shown higher statistically significant values (*p* < 0.05) in RCC tumors and is a sufficient discriminatory radiomic marker
Zhou et al. [83]	Benign vs. RCC (N = 192) At least one phase of CECT	Axial Multichannel (RGB) 2D ROI images	ROI: 2D Radiomics: TL of pretrained ImageNet Classification: InceptionV3, softmax, 5-fold CV	Acc: 0.97 Sen: 0.95 Spe: 0.97 AUC: —	Deep learning can potentially be used to identify malignant renal tumors using deep transfer learning
Kim et al. [61]	Renal Cysts vs. RCC (N = 501) Unenhanced CT	1st-Order Statistics: 3 – entropy – kurtosis – MGLA	ROI: 2D Radiomics: TexRAD software Classification: LR (Threshold)	Acc: 0.84 Sen: 0.81 Spe: 0.89 AUC: range (0.89–0.92)	Entropy ≥ 4 differentiated RCC from benign renal tumors (AUC = 0.89). A better AUC of 0.92 is obtained using a combined model
Nie et al. [84]	AMLwvf vs. RCC (N = 99) Multiphasic CECT	Shape: 2 – Phase 2: 1 – Phase 3: 1 1st-Order Statistics: 3 – Phase 2: 1 – Phase 3: 2 2nd-Order Statistics: 9 – Phase 2: 3 (GLCM), 3 (GLDM), 1 (GLRLM) – Phase 3: 2 (GLRLM)	ROI: 3D Radiomics: RadCloud software, LASSO Classificiation: Nomogram, Rad-score ≥ 0.017, 20% validation	Acc: 0.84 Sen: 0.85 Spe: 0.83 AUC: 0.85	Radiomics of multiphasic CECT distinguish AMLwvf from ccRCC. A combined model that integrates clinical factors, with a Nomo-score ≥ 1.451, provided higher diagnostic performance (Acc = 0.89, AUC = 0.95)
Tang et al. [57]	AMLwvf vs RCC (N = 115) Multiphasic CECT	1st-Order Statistics: 24 2nd-Order Statistics: 52 – GLCM: 23 – GLRLM: 11 – GLSZM: 13 – NGTDM: 5 Higher-Order Statistics: 120 – LTE: 120	ROI: 2D Radiomics: In-house software, 100% Data Augmentation, LASSO Classification: LR	Acc: range (0.8–0.92) Sen: — Spe: — AUC: range (0.67–0.92)	Integrating different radiomic markers is potentially helpful in distinguishing AMLwvf from RCC renal tumors
Lee et al. [53] (Study 1)	AMLwvf vs. ccRCC (N = 50) Multiphasic CECT	1st-Order Statistics: 3 2nd-Order Statistics: 1 – GLCM: 1	ROI: 2D Radiomics: ImageJ software, ReliefF Classification: *k*NN, SVM, 5-fold CV	Acc: 0.72 Sen: 0.72 Spe: 0.73 AUC: 0.75	Proper selection and integration of optimal radiomic markers and machine learning-based classifiers could sufficiently differentiate between AMLwvf and ccRCC
Lee et al. [85] (Study 2)	AMLwvf vs. ccRCC (N = 80) Multiphasic CECT	Shape: 7 1st-Order Stats: 18 2nd-Order Stats: 53 – GLCM: 14 – GLDM: 22 Higher-Order Stats: 10 – LBP: 10 1000–4000 dimensional deep markers extracted from ImageNet pretrained models (GoogleNet, AlexNet, VGGNet, and ResNet) with image patches of small renal masses.	ROI: 2D Radiomics: ImageNet pretrained Classification: RF, k-fold CV	Acc: range (0.75–0.77) Sen: range (0.73–0.79) Spe: range (0.75–0.77) AUC: range (0.79–0.82)	A combined model integrating hand-crafted with deep radiomic markers provided an enhanced diagnostic performance than individual models and thus; has the potential to distinguish AMLwvf from ccRCC
Feng et al. [54] (Study 1)	AMLwvf vs. RCC (N = 58) Multiphasic CECT	1st-Order Statistics: 8 2nd-Order Statistics: 3 – GLCM: 3	ROI: 2D Radiomics: CT Kinetics software, SMOTE, RFE Classification: SVM, 5-fold CV	Acc: 0.94 Sen: 0.88 Spe: 1.00 AUC: 0.96	Combination of SVM, RFE, and SMOTE can help selecting optimal radiomics that could accurately distinguish AMLwvf from RCC
Yan et al. [55]	AMLwvf vs. ccRCC and paRCC (N = 50) Multiphasic CECT	1st-Order Statistics: 11 2nd-Order Statistics: 220 – GLCM: 220	ROI: 2D Radiomics: MaZda software, NDA Classification: *k*NN, ANN, 5-fold CV	Acc: range (0.97–1.00) Sen: — Spe: — AUC: —	Optimal radiomics of multiphasic CECT images can potentially be used to discriminate between AMLwvf, ccRCC, and paRCC
Hodgdon et al. [14]	AMLwvf vs. RCC (N = 100) Unenhanced CT	1st-Order Statistics: 2 2nd-Order Statistics: 7 – GLCM: 5 – GLRLM: 2	ROI: 2D Radiomics: MaZda software Classification: SVM, 10-fold CV	Acc: range (0.83–0.91) Sen: — Spe: — AUC: range (0.73–0.90)	Radiomic markers of unenhanced CT images can differentiate between AMLwvf and RCC
Tanaka et al. [62]	Benign vs. Malignant (N = 168) Multiphasic CECT	2D ROI images around the lesion (299 × 299) Data were augmented using rotation, mirroring, and addition of Gaussian noise techniques.	ROI: 2D Radiomics: Inception-V3 CNN Classification: Inception-V3 CNN, 20% testing	Acc: range (0.41–0.88) Sen: range (0.29–0.96) Spe: range (0.33–1.00) AUC: range (0.49–0.85)	Deep learning can be used to identify malignant tumors, especially in Phase 2 CECT
Kunapuli et al. [86]	Benign vs. Malignant (N = 150) Multiphasic CECT	1st-Order Statistics: 2 2nd-Order Statistics: 8 – GLCM: 7 – GLDM: 1	ROI: 2D/3D Radiomics: In-house software, RFE Classification: RFGB, 10-fold CV	Acc: 0.82 Sen: — Spe: — AUC: 0.83	RFGB machine learning classifier and radiomic markers have the potential to identify the malignancy status of renal tumors
Yap et al. [59]	Benign vs. Malignant (N = 735) Multiphasic CECT	Total: top 10% (79) Shape: — 1st-Order Statistics: — 2nd-Order Statistics: — – GLCM: — – GLDM: — Higher-Order Statistics: — – FFT: —	ROI: 3D Radiomics: PyRadiomics, Gini Classification: RF, AdaBoost, 10-fold CV	Acc: — Sen: — Spe: — AUC: range (0.65–0.75)	Combining shape and texture radiomic markers of multiphasic CECT can improve the overall diagnostic performance
Ma et al. [56] (Study 1)	AMLwvf vs. ccRCC (N = 84) Multiphasic CECT	Total: 6 1st-Order Statistics: — 2nd-Order Statistics: — – GLCM: —	ROI: 3D Radiomics: AI-Kit, LASSO Classification: LR, 30% testing	Acc: — Sen: — Spe: — AUC: range (0.83–0.93)	Combined model integrating radiomics from different phases of CECT enhanced the final diagnostic performance when compared with individual models as well as unenhanced CT
Ma et al. [87] (Study 2)	AMLwvf vs. ccRCC (N = 230) Multiphasic CECT	Total: 396 Shape: — 1st-Order Statistics: — 2nd-Order Statistics: — – GLCM: — – GLRLM: — – GLSZM: —	ROI: 3D Radiomics: AI-Kit, LASSO Classification: LR, 30% validation	Acc: range (0.69–0.80) Sen: range (0.66–0.79) Spe: range (0.76–0.85) AUC: range (0.74–0.89)	The perirenal model that extracts radiomic markers from Phase 3 CECT is superior to other phases to distinguish between AMLwvf and ccRCC
Nassiri et al. [58]	Benign vs. Malignant (N = 684) Multiphasic CECT	Shape: — 1st-Order Statistics: — 2nd-Order Statistics: — – GLCM: — – GLDM: — – GLRLM: — – NGTDM: — – GLSZM: — Higher-Order Statistics: — – DCT: — – FFT: — – LTE: —	ROI: 3D Radiomics: Pyradiomics, Gini Classification: RF, Adaboost, and 10-fold CV	Acc: range (0.74–0.79) Sen: range (0.73–0.80) Spe: 0.75 AUC: range (0.77–0.84)	Radiomic markers of Phase 3 CECT can sufficiently identify malignancy status. When combined, some clinical factors can improve the overall diagnostic performance
Zabihollahy et al. [66]	Benign vs. RCC (N = 315) Multiphasic CECT	2D ROI images around the tumor (512 × 512)	ROI: 2D Radiomics: CNN Classification: CNN, MJV, 50% testing	Acc: range (0.77–0.84) Sen: range (0.84–0.92) Spe: range (0.26–0.52) AUC: —	Semiautomated CNN showed the highest diagnostic performance in distinguishing RCC from benign renal tumors using CECT
Uhlig et al. [36] (Study 1)	Benign vs. Malignant (N = 94) Phase 3 CECT	Total: 120 Shape: — 1st-Order Statistics: — 2nd-Order Statistics: — – GLCM: — – GLDM: — – GLRLM: — – NGTDM: — – GLSZM: —	ROI: 3D Radiomics: PyRadiomics, RFE, and LR Classification: RF, 10-fold CV	Acc: 0.84 Sen: 0.88 Spe: 0.67 AUC: 0.83	Radiomic markers derived from Phase 3 CECT can successfully differentiate benign from malignant renal tumors using RF machine learning classifier
Li et al. [63] (Study 1)	ONC vs. chrRCC (N = 61) Multiphasic CECT	1st-Order Statistics: 3 – Phase 2 and 3: 2 – Phase 4: 1 2nd-Order Statistics: 5 – Phase 2 and 3: 3 (GLCM) 3 (GLCM) – Phase 2 and 3: 2 (wavelet)	ROI: 3D Radiomics: PyRadiomics, LASSO Classification: SVM, 5-fold CV	Acc: 0.95 Sen: 0.99 Spe: 0.80 AUC: 0.85	Radiomics derived from multiphasic CECT can accurately differentiate chrRCC from ONC using SVM
Li et al. [64] (Study 2)	ONC vs. ccRCC (N = 122) Multiphasic CECT	1st-Order Statistics: 5 – Phase 2: 2 – Phase 3: 3 2nd-Order Statistics: 6 – Phase 2: 2 (GLCM), 1 (GLSZM) – Phase 3: 1 (GLCM) – Phase 4: 2 (GLCM)	ROI: 3D Radiomics: PyRadiomics, LASSO, and LR Classification: Nomogram, Rad-score, and 30% validation	Acc: 0.81 Sen: 0.86 Spe: 0.83 AUC: 0.84	Radiomics of multiphasic CECT can differentiate ONC from ccRCC. By integrating clinical factors, enhanced diagnosis is obtained (Acc = 0.87, Sen = 0.86, Spe = 0.87, and AUC = 0.90)
Li et al. [65] (Study 3)	ONC vs. chrRCC (N = 141) Multiphasic CECT	1st-Order Statistics: 5 – Phase 2: 1 – Phase 3: 2 – Phase 4: 2 2nd-Order Statistics: 7 – Phase 2: 2 (GLCM), 1 (GLSZM) – Phase 3: 2 (GLCM) – Phase 4: 2 (GLCM)	ROI: 3D Radiomics: PyRadiomics, LASSO, LR Classification: Nomogram, Rad-score ≥ −0.55, and 40% validation	Acc: 0.91 Sen: 0.84 Spe: 0.95 AUC: 0.96	Radiomics of multiphasic CT can differentiate ONC from chrRCC. Clinical factors, when combined, with a Nomo-score ≥0.19 can enhance the diagnosis (Acc = 0.95, Sen = 0.90, Spe = 0.97, and AUC = 0.99)
**Main Goal(s): Malignant Subtyping**					
Uhlig et al. [70] (Study 2)	Subtyping (N = 201) Phase 3 CECT	Total: 127 Shape: — 1st-Order Statistics: — 2nd-Order Statistics: — – GLCM: — – GLDM: — – GLRLM: — – NGTDM: — – GLSZM: —	ROI: 3D Radiomics: PyRadiomics, SMOTE, and RFE Classification: XGBoost, RF, and 10-fold CV	Acc: range (0.54–0.92) Sen: range (0.05–0.80) Spe: range (0.41–0.97) AUC: range (0.45–0.85)	Radiomic markers extracted from Phase 3 CECT along with machine learning classifiers, can help distinguish different subtypes. Differentiation of ONCs remains challenging
Uhm et al. [88]	Subtyping (N = 308) Multiphasic CECT	3D channel image of size 224 × 224 cropped from the largest segmented tumor at phases 2, 3, and 4 ResNet-101 was initialized with weights obtained from a pretrained ImageNet.	ROI: 2D Radiomics: CNN Classification: ResNet-101, 16% validation, and (N = 184) external test	Acc: 0.72 Sen: range (0.60–0.89) Spe: range (0.87–0.97) AUC: 0.89	Deep learning outperformed radiological diagnosis of renal tumors using multiphasic CECT
Kocak et al. [89]	RCC Subtyping (N = 68) Phases 1 and 2 of CECT	1st-Order Statistics: 9 – Phase 1: 5 – Phase 2: 4 2nd-Order Statistics: 16 – Phase 1: 3 (GLCM) – Phase 2: 13 (GLCM) Higher-Order Statistics: 5 – Phase 1: 4 (wavelet), 1 (autoagressive)	ROI: 2D Radiomics: wrapper, Nested 10-fold CV Classification: SMOTE, SVM, and ANN; (N = 26) external test	Acc: range (0.69–0.85) Sen: range (0.69–0.71) Spe: 1.00 AUC: —	Combined radiomics extracted from phases 1 and 2 (Phase 2 is superior) can distinguish RCC major subtypes using machine learning. Distinguishing ccRCC, paRCC, chrRCC remains challenging
Zhang et al. [35]	RCC Subtyping (N = 127) Multiphasic CECT	1st-Order Statistics: 4 – mean – STD – entropy – kurtosis	ROI: 2D Radiomics: TexRAD software, Catboost Classification: SVM, 10-fold CV	Acc: range (0.78–0.87) Sen: range (0.87–0.89) Spe: 0.92 AUC: range (0.94–0.96)	Radiomic markers of Phase 2 CECT have the potential for RCC subtyping using SVM
Chen et al. [71]	RCC Subtyping (N = 197) Multiphasic CECT	2nd-Order Statistics: 9 – Phase 1: 3 (GLCM), 1 (GLRLM) – Phase 2: 1 (GLCM) – Phase 3: 1 (GLCM), 1 (GLSZM) – Phase 4: 2 (GLCM)	ROI: 3D Radiomics: PyRadiomics, LASSO Classification: SMOTE, LR	Acc: range (0.82–0.88) Sen: range (0.81–0.89) Spe: range (0.81–0.88) AUC: range (0.86–0.90)	Second-order radiomics integrated with nontexture markers of Phase 3 CECT provide the best RCC subtyping performance (AUC = 0.9)
Deng et al. [34] (Study 2)	RCC Subtyping (N = 298) Phase 3 CECT	1st-Order Statistics: 4 – mean – entropy – kurtosis – skewness	ROI: 2D Radiomics: TexRAD software, LSSF Classification: LR	Acc: 0.47 Sen: 0.31 Spe: 0.86 AUC: range (0.80–0.84)	Entropy had shown higher statistically significant values in ccRCC (*p* < 0.05) with high values being correlated with RCC’s high grade
**Main Goal(s): Benign vs. Malignant and Malignant Subtyping**					
Shehata et al. [68]	AML vs. RCC ccRCC vs. nccRCC (N = 140) Multiphasic CECT	Shape: 70 – 70 SHREs 1st-Order Statistics: 16 2nd-Order Statistics: 6 – GLCM: 6 Functional: 2 – wash-in slope – wash-out slope	ROI: 3D Radiomics: In-house Software Classification: MLP-ANN, k-fold, 10-fold CV	Acc: range (0.79–0.98) Sen: range (0.89–0.95) Spe: range (0.91–1.00) AUC: –	A MLP-ANN diagnostic model integrating shape, texture, and functional radiomic-based markers can identify malignant renal tumors as well as their subtypes.
Yu et al. [67]	Benign vs. Malignant RCC Subtyping (N = 119) Phase 3 CECT	1st-Order Statistics: 14 2nd-Order Statistics: 20 – GLCM: 5 – GLRLM: 11 – GLGM: 4 Higher-Order Statistics: 9 – LTE: 9	ROI: 2D Radiomics: In-house Software Classification: SVM, 5-fold CV	Acc: — Sen: — Spe: — AUC: range (0.86–0.92)	Machine learning and 1st-Order radiomic markers (e.g., skewness, kurtosis, and median) demonstrates high diagnostic performance of different renal tumors’ types.
Varghese et al. [69]	Benign vs. Malignant RCC Subtyping (N = 174) Multiphasic CECT	1st-Order Stats: 8 2nd-Order Stats: 20 – GLCM: 13 – GLDM: 7 Higher-Order Stats: 3 – FFT: 3	ROI: 3D Radiomics: PyRadiomics Classification: Stepwise LR (Statistical analysis only)	Acc: — Sen: — Spe: — AUC: range (0.80–0.98)	With a significance level (*p* < 0.05), various radiomic markers are helpful in discriminating benign from malignant renal tumors as well as RCC subtypes
Cui et al. [52]	AMLwvf vs. RCC RCC Subtyping (N = 168) Multiphasic CECT	AMLwvf vs. RCC: 17 AMLwvf vs. ccRCC: 21 AMLwvf vs. nccRCC: 12 1st-Order Statistics: — 2nd-Order Statistics: — – GLCM: — – GLRLM: — – GLSZM: — – NGTDM: — – GLDM: —	ROI: 3D Radiomics: PyRadiomics, RFE Classification: SMOTE, SVM, and 5-fold CV	Acc: range (0.84–0.93) Sen: range (0.83–0.95) Spe: range (0.85–0.96) AUC: range (0.89–0.97)	Machine learning-based radiomics techniques are comparable to radiological assessment and can precisely distinguish AMLwvf from RCC and its subtypes
**Main Goal(s): Malignant Grading**					
Sun et al. [90]	ccRCC Grading (N = 227) Multiphasic CECT	1st-Order Statistics: 1 – Phase 2 and 3: RMS 2nd-Order Statistics: 6 – Phase 2 and 3: 1 (GLCM), 3 (GLSZM), 2 (GLRLM)	ROI: 2D Radiomics: RadCloud software, LASSO, and ICC Classification: SVM, 20% validation	Acc: 0.87 Sen: 0.83 Spe: 0.89 AUC: 0.91	Radiomics extracted and combined from phases 2 and 3 of CECT have the potential to successfully grade ccRCC renal tumors using SVM
Feng et al. [37] (Study 2)	ccRCC Grading (N = 131) Multiphasic CECT	1st-Order Statistics: 5 – mean – entropy – STD – skewness – kurtosis	ROI: 2D Radiomics: In-house software Classification: *t*-test (Statistical analysis only)	Acc: range (0.70–0.79) Sen: range (0.76–0.95) Spe: range (0.54–0.77) AUC: range (0.74–0.83)	Statistically significant (*p* < 0.05) radiomics markers, such as entropy, STD, and kurtosis are superior to grade ccRCC renal tumors
Shu et al. [38] (Study 1)	ccRCC Grading (N = 260) Phases 2 and 3 of CECT	Shape: 5 – Phase 2: 1 – Phase 3: 4 1st-Order Statisitcs: 9 – Phase 2: 3 – Phase 3: 6 2nd-Order Statistics: 21 – Phase 2: 2 (GLCM), 3 (GLSZM), 2 (GLRLM) – Phase 3: 3 (GLCM), 8 (GLSZM), 3 (GLRLM)	ROI: 3D Radiomics: In-house software, ICC, and LASSO Classification: LR, 5-fold CV	Acc: range (0.72–0.78) Sen: range (0.60–0.69) Spe: range (0.83–0.84) AUC: range (0.77–0.82)	Combined radiomic markers extracted from phases 2 and 3 of CECT are sufficient for ccRCC grading
Shu et al. [91] (Study 2)	ccRCC Grading (N = 271) Phases 2 and 3 of CECT	1st-Order Statistics: 4 – Phase 2: 1 – Phase 3: 3 2nd-Order Statistics: 8 – Phase 2: 1 (GLCM), 3 (GLRLM) – Phase 3: 2 (GLCM), 1 (GLRLM), 1 (GLSZM)	ROI: 3D Radiomics: RadCloud software, ICC, and LASSO Classification: SVM, RF, and MLP; 40% validation	Acc: range (0.92–0.94) Sen: range (0.92–0.97) Spe: range (0.86–0.95) AUC: range (0.96–0.98)	Combined radiomic markers extracted of phases 2 and 3 of CECT along with machine learning could be sufficiently used for ccRCC grading
Ding et al. [72]	ccRCC Grading (N = 114) Phases 2 and 3 of CECT	2nd-Order Statistics: 4 – Phase 2: 1 (GLRLM) – Phase 3: 3 (GLCM)	ROI: 2D Radiomics: IBEX software, LASSO Classification: LR, (N = 92) external test	Acc: — Sen: — Spe: — AUC: ≥0.67	Radiomic markers of phases 2 and 3 of CECT are helpful in ccRCC grading
Bektas et al. [74]	ccRCC Grading (N = 54) Phase 3 CECT	2nd-Order Statistics: 8 – Phase 3: 5 (GLCM), 3 (GLRLM) Higher-Order Stats: 5 – Phase 3: 4 (wavelet), 1 (gradient)	ROI: 2D Radiomics: MaZda software, wrapper, and Nested 10-fold CV Classification: SVM	Acc: 0.85 Sen: 0.91 Spe: 0.80 AUC: 0.86	SVM machine learning classifier and radiomic markers of Phase 3 CECT can be used in ccRCC grading
Lin et al. [75]	ccRCC Grading (N = 232) Multiphasic CECT	1st-Order Stats: 6 2nd-Order Stats: 16 – GLCM: 4 – GLDM: 4 – GLRLM: 4 – NGTDM: 1 – GLSZM: 3	ROI: 2D Radiomics: PyRadiomics Classification: GBDT, 5-fold CV	Acc: 0.74 Sen: 0.14 Spe: 0.88 AUC: 0.87	Using machine learning, combined radiomic markers from phases 1, 2, and 3 of CECT can sufficiently grade ccRCCs
He et al. [80]	ccRCC Grading (N = 227) Multiphasic CECT	1st-Order Statistics: 6 – Phase 2: 4 – Phase 3: 2 2nd-Order Statistics: 14 – Phase 2: 7 (GLCM), 3 (GLRLM) – Phase 3: 3 (GLCM), 1 (GLRLM) Higher-Order Statistics: 9 – Phase 2: 1 (gradient), 4 (wavelet) – Phase 3: 1 (gradient), 3 (wavelet)	ROI: 2D Radiomics: MaZda software, LASSO Classification: ANN, 15% validation, 15% testing, and 10-fold CV	Acc: range (0.91–0.94) Sen: — Spe: — AUC: —	Combined radiomic markers of phases 2 and 3 of CECT have the potential for RCC grading using ANN
Momenian et al. [76]	ccRCC Grading (N = 103) Phases 2 and 3 of CECT	1st-Order Statistics: 18 – Phase 1: 6 – Phase 2: 6 – Phase 3: 6 2nd-Order Statistics: 93 – Phase 1: 20 (GLCM), 11 (GLRLM) – Phase 2: 20 (GLCM), 11 (GLRLM) – Phase 3: 20 (GLCM), 11 (GLRLM)	ROI: 2D Radiomics: In-house software Classification: RF, 10-fold CV	Acc: 0.97 Sen: — Spe: — AUC: —	First-order radiomic markers extracted from Phase 2 CECT showed the best diagnostic performance in ccRCC grading using the RF classifier when compared with 2nd-order radiomic markers alone as well as combined radiomic markers.
Yin et al. [73]	ccRCC Grading (N = 78) Phase 2 CECT	2nd-Order Statistics: 10 – Phase 2: 7 (GLCM), 3 (GLRLM)	ROI: 2D Radiomics: AI-Kit, ICC, SMOTE Classification: SVM, 10-fold CV, 32% testing	Acc: 0.88 Sen: 0.80 Spe: 0.90 AUC: 0.86	2nd-Order radiomic markers of Phase 2 CECT provided the highest ccRCC grading performance using SVM
Lai et al. [77]	ccRCC Grading (N = 137) Multiphasic CECT	Shape: 5 – Phase 1: 5 1st-Order Statistics: 5 – Phase 1: 5 mean, median, RMS, 10th Pctl, 90th Pctl	ROI: 2D Radiomics: PyRadiomics, CMIM, and SMOTE Classification: Bagging, 10-fold CV	Acc: — Sen: — Spe: — AUC: 0.75	Shape and 1st-Order radiomics extracted from Phase 1 CECT along with a Bagging classifier provided the highest ccRCC grading performance (AUC = 0.75)
Yi et al. [79]	ccRCC Grading (N = 264) Phases 1 and 3 of CECT	1st-Order Statistics: 6 – Phase 1: 6 2nd-Order Statistics: 9 – Phase 1: 9 (GLRLM) Higher-Order Stats: 4 – Phase 1: 4 (wavelet)	ROI: 2D Radiomics: MaZda software, ICC, LASSO Classification: SVM, 25% validation	Acc: 0.90 Sen: 0.94 Spe: 0.89 AUC: 0.91	Radiomic markers of Phase 1 CECT can successfully grade ccRCCs using SVM (AUC = 0.91)
Xu et al. [81]	ccRCC Grading (N = 706) Phase 2 CECT	2D ROI images (224 × 224 × 3) as input to VGG-16 pretrained on ImageNet for segmentation. Self-supervised pretraining using RegNetY400MF, RegNetY800MF, SE-ResNet50, ResNet101, and Ensamble.	ROI: 2D Radiomics: VGG-16 Classification: Ensamble, 16% validation	Acc: 0.82 Sen: 0.86 Spe: 0.75 AUC: 0.88	Deep learning applied on Phase 2 CECT images has the potential to grade ccRCC renal tumors with an AUC of 0.88 using the combined (Ensamble) model outperforming all other individual models.
Luo et al. [78]	ccRCC Grading (N = 177) Multiphasic CECT	Shape: 7 – Phase 1 and 4: 7 1st-Order Statistics: 4 – Phase 1 and 4: 4 median, RMS, 10th Pctl, 90th Pctl	ROI: 3D Radiomics: PyRadiomics, CIFE, and SMOTE Classification: RF, 5-fold CV	Acc: 0.81 Sen: 0.67 Spe: 0.87 AUC: 0.87	Shape and 1st-Order radiomics extracted from phase 1 and 4 of CECT along with an RF classifier demonstrated the highest diagnostic performance in ccRCC grading (AUC = 0.87)
**Main Goal(s): Malignant Grading and Staging**					
Demirjian et al. [39]	ccRCC Grading ccRCC Staging (N = 587) Multiphasic CECT	Grading: – 1st-Order Statistics: intensity – 2nd-Order Statistics: 2D GLCM, 3D GLCM, 3D GLRLM Staging: – 2nd-Order Statistics: 2D GLCM, 3D GLCM, 2D GLDM, 3D GLDM	ROI: 3D Radiomics: In-house software, ICC, and Gini index Classification: RF, 32% testing	Acc: — Sen: — Spe: — AUC: 0.73 and 0.77	Radiomic markers of multiphasic CECT have the potential to grade and stage ccRCCs using RF (AUC = 0.73 and 0.77)

Notes: Phase 1, unenhanced or precontrast; Phase 2, arterial or corticomedullary; Phase 3, portal venous or nephrographic; Phase 4, delayed or excretory; SFS, sequential feature selection; TL, transfer learning; GTf, Google Tensorflow; LSSF, Laplacian spatial scaling factor; MGLA, mean gray-level attenuation; LTE, Law’s texture energy; LBP, local binary pattern; SMOTE, synthetic minority oversampling technique; NDA, nonlinear discriminant analysis; RFE, recursive feature elimination; RFGB, relational functional gradient boosting; FFT, fast Fourier transform; DCT, discrete cosine transform; MJV, majority voting; ICC, interclass correlation coefficient; MLP-ANN, multilayer perceptron artificial neural networks; RMS, root mean squared; IBEX, Imaging Biomarker EXplorer; GBDT, gradient boosting decision tree; Pctl, percentile; CMIM, conditional mutual information maximization; RMAD, robust mean absolute difference; CIFE, supervised feature selection methods.

**Table 2:** Summary of last decade MRI-based studies to early diagnose renal tumors.

Study	Data	Radiomics	Methods	Results	Findings
Xu et al. [29]	Benign vs. Malignant (N = 217) T2-weighted MRI and DW-MRI	Shape: — 1st-Order Statistics: 1 2nd-Order Statistics: 7 – GLCM: 2 – GLRLM: 3 – GLSZM: 1 – GLDM: 1	ROI: 2D Radiomics: PyRadioimcs, LASSO Classification: ResNet-18, RF, 10-fold CV	Acc: range (0.70–0.82) Sen: range (0.81–0.94) Spe: range (0.33–0.92) AUC: range (0.74–0.93)	Combined radiomic markers of multimodal MRIs can sufficiently identify the malignancy status of renal tumors by utilizing handcrafted-based RF or DL-based classification models
Oostenburgge et al. [30]	ONC vs. RCC (N = 39) 3D ADCs of DW-MRIs	1st-Order Statistics: 2 – entropy – STD Tumor volume	ROI: 2D Radiomics: In-house software Classification: ICC, *U*-test, ROC, and LR	Acc: 0.87 Sen: 0.86 Spe: 0.84 AUC: 0.91	Radiomics extracted from 3D ADCs such as standard deviation and entropy can discriminate ONC from RCC when combined with tumor volume and gender
Li et al. [31]	Benign vs. Malignant (N = 92) 3D ADCs of DW-MRIs	1st-Order Statistics: 6 – mean – median – STD – entropy – 75th pctl – 90th pctl	ROI: 2D Radiomics: PASW IBM software Classification: ANOVA, *t*-test, and ROC (Statistical analysis only)	Acc: 0.82 Sen: 0.80 Spe: 0.86 AUC: 0.85	Radiomic markers extracted from 3D ADCs of DW-MRIs are significantly higher (*p* < 0.05) in malignant than benign tumors
Razik et al. [23]	Benign vs. Malignant (N = 54) Multiparametric MRIs	1st-Order Statistics: 2 – mean – MPP	ROI: 2D Radiomics: TexRAD software Classification: *U*-test, ROC (Statistical analysis only)	Acc: range (0.79–0.95) Sen: range (0.71–0.97) Spe: range (0.80–1.00) AUC: range (0.89–0.94)	MPP and the mean value can distinguish RCC from AML as well as RCC from ONC with an AUC of 0.89 and 0.94 at $b_{500}$ s/mm^2^ and $b_{1000}$ s/mm^2^ of DW-MRI, respectively
Nikpanah et al. [92]	ONC vs. ccRCC (N = 243) T2-weighted MRI and multiphasic CEMRI	Local ROI patch was automatically extracted, with a size of 100 × 100 mm. RGB image patches were resized to 224 × 224 to fit the pretrained AlexNet configuration.	ROI: 2D Radiomics: AlexNet CNN Classification: AlexNet, 5-fold	Acc: 0.81 Sen: 0.88 Spe: 0.75 AUC: 0.90	Using multiphasic MRIs, DL-based system can provide high diagnostic performance that differentiates ONC from ccRCC renal tumors
Arita et al. [93]	AML vs. nccRCC (N = 106) 3D ADCs of DW-MRIs	1st-Order Statistics: 7 2nd-Order Statistics: 13 – GLCM: 4 – GLRLM: 4 – GLSZM: 4 – GLDM: 1	ROI: 3D Radiomics: LIFEx software Classification: RF, 37% validation	Acc: 0.77 Sen: 0.87 Spe: 0.69 AUC: 0.82	Long-zone high grey-level emphasis is the most informative radiomic marker to distinguish between AML and nccRCC using an RF classifier with (AUC = 0.82)
Gunduz et al. [94]	ONC vs. chrRCC (N = 14) 3D ADCs of DW-MRIs	1st-Order Statistics: 1 – squared root of mean ADC 2nd-Order Statistics: 5 – GLRLM: 5	ROI: 3D Radiomics: PyRadiomics Classification: ICC, ROC (Statistical analysis only)	Acc: 0.86 Sen: 0.88 Spe: 0.83 AUC: 0.94	Squared root of mean ADC and GLRLM radiomic markers of ADC maps can sufficiently differentiate between ONC and chrRCC
Matsumoto et al. [32]	AML vs. ccRCC (N = 122) 3D ADCs of DW-MRIs	1st-Order Statistics: 3 – mean ADC – skewness – entropy 2nd-Order Statistics: 9 – GLCM: 3 – GLRLM: 4 – GLZLM: 1 – GLDM: 1	ROI: 3D Radiomics: LIFEx software Classification: RF, 32% validation	Acc: — Sen: — Spe: — AUC: 0.87	Mean ADC, grey-level run emphasis, and long-run low grey-level, are the most dominant and important radiomic markers in distinguishing AML from ccRCC with an AUC of 0.87
Hoang et al. [96] (Study 1)	Benign vs. RCC (N = 212) Multiphasic CEMRI	1st-Order Statistics: 4 – mean – STD – skewness – kurtosis	ROI: 2D Radiomics: In-house software Classification: RF, 50% validation	Acc: 0.84 Sen: — Spe: — AUC: —	Using an RF classification model, first-order radiomic markers of multiphasic CEMRI have the potential to identify RCC renal tumors
**Main Goal(s): Benign vs. Malignant and Malignant Subtyping**					
Hoang et al. [33] (Study 2)	ONC vs. ccRCC and paRCC ccRCC vs. paRCC (N = 140) Multiphasic CEMRI	1st-Order Statistics: 5 2nd-Order Statistics: 40 – GLCM: 9 – GLRLM: 13 – GLSZM: 13 – NGTDM: 5	ROI: 2D Radiomics: Radiomics-developed package, LASSO Classification: RF, 5-fold CV	Acc: range (0.78–0.79) Sen: range (0.67–0.70) Spe: range (0.86–0.89) AUC: —	First-order radiomic markers are important for identifying the malignancy status, while adding second-order markers helps in RCC subtyping
**Main Goal(s): Malignant Grading**					
Sun et al. [40]	ccRCC Grading (N = 45) SW-MRI	Shape: 2 2nd-Order Statistics: 8 – GLCM: 2 – GLRLM: 1 – GLSZM: 2 – GLDM: 3	ROI: 2D Radiomics: AI-Kit, ICC, *U*-test Classification: LR, 30% validation	Acc: 0.77 Sen: 0.81 Spe: 0.71 AUC: 0.81	Radiomic markers of SW-MRI can reliably differentiate low-grade from high-grade ccRCC
Chen et al. [41]	ccRCC Grading (N = 99) Phase 2 CEMRI	1st-Order Statistics: 2 – entropy – kurtosis 2nd-Order Statistics: 4 – GLCM: 1 – GLRLM: 3	ROI: 2D Radiomics: MaZda, ICC, RF Classification: MLP-ANN, 30% validation	Acc: 0.86 Sen: 0.73 Spe: 0.94 AUC: 0.76	First- and second-order radiomic markers of Phase 2 CEMRI along with MLP-ANN classification model have the potential to grade ccRCC
Choi et al. [95]	ccRCC Grading (N = 364) T2-weighted MRI and multiphasic CEMRI	Shape: 5 2nd-Order Statistics: 15 – GLDZM: 15	ROI: 3D Radiomics: Radiomics- developed package, ANOVA Classification: RF, 30% testing	Acc: 0.98 Sen: 0.72 Spe: 0.95 AUC: 0.89	Proper selection and integration of optimal radiomic markers of MRIs can potentially help grade ccRCCs
**Main Goal(s): Malignant Subtyping and Grading**					
Goyal et al. [22]	ccRCC vs. nccRCC ccRCC grading (N = 34) Multiparametric MRIs	1st-Order Statistics: 6 – mean – STD – MPP – entropy – skewness – kutrosis	ROI: 2D Radiomics: TexRAD Classification: *U*-test, ROC (Statistical analysis only)	Subtyping: AUC range (0.81–0.91) Grading: AUC range (0.82–0.89)	Multiple first-order radiomic markers of multiparametric MRIs are beneficial tools in both subtyping and grading of renal tumors

Notes: LASSO, least absolute shrinkage and selection operator; ADCs, apparent diffusion coefficients; SW-MRI, susceptibility-weighted MR imaging; LIFEx, local image feature extraction; ANOVA, analysis of variance; MPP, mean positive pixels; MLP-ANN, multilayer perceptron artificial neural network.

**Table 3:** Summary of the last decade’s CT- and/or MRI-based studies for predicting/assessing patient outcomes (e.g., treatment response, recurrence, and overall survival (OS), and progression-free survival (PFS)).

Study	Main Goal	Radiomics	Methods	Results	Findings
Bharwani et al. [43]	To find the radiomic markers extracted from diffusion-weighted MR (DW-MR) and dynamic contrast-enhanced MR (DCE-MR) images that correlate with responses to neoadjuvant sunitinib therapy, in particular overall survival (OS), in metastatic renal cell carcinoma (mRCC) patients (N = 20)	Shape: – median tumor volume 1st-Order Statistics (DW-MRI): – mean ADC – AUC_low_ (ADC 25th Pctl.) – kurtosis – skewness Functional DCE-MRI: – max signal intensity – wash-in rate	2D and 3D ROIs KM (Statistical analysis only)	Acc: – Sen: – Spe: – AUC: –	Patients with a tumour volume < median at baseline had a prolonged OS. A greater than median increase in AUC_low_ of ADCs indicates reduced OS while a decrease in AUC_low_ indicates a prolonged OS in mRCC. A positive correlation between mean ADC was found between the primary tumor and metastases
Antunes et al. [44]	To find the optimal radiomic markers on an integrated positron emission tomography (PET)/MRI that best describe early treatment response/changes in advanced mRCC undergoing sunitinib therapy (N = 2)	Total: 66 (raw T2w signal, postprocessed T2w, 30 postprocessed T2w textures, raw ADC map, 30 ADC textures, standard uptake value (SUV), and 2 PET textures)	2D ROI Scoring function	Acc: – Sen: – Spe: – AUC: –	SUV from PET, T2w difference average from T2w, and ADC energy from DW-MRI ADC maps are ranked highest for reproducibility and for capturing treatment related changes/response
Lubner et al. [50]	To determine the radiomic-based texture markers extracted from CECT images on phases 1 and 3 of RCCs patients that are correlated with the histological finding and treatment response (N = 157)	1st-Order Statistics: 6 – mean – STD – MPP – entropy – skewness – kurtosis	2D ROI SLR, LR CPHR, KM (statistical analysis only)	Acc: – Sen: – Spe: – AUC: –	1st-Order texture markers (entropy, STD, and MPP) extracted from phases 1 and 3 of CECT are correlated with histologic type, nuclear grade, and clinical outcomes (time to recurrence and OS) in patients with RCC
Boos et al. [45]	To assess the ability of mean and median intensity attenuation (HU) using CECT images for predicting treatment response (response, stable, and progression) in patients with RCC tumors who received targeted therapy, namely VEGFR TKI (N = 19)	Functional: – median HU – mean HU	2D ROI WRST (statistical analysis only)	Acc: range (0.63 - 0.79) Sen: – Spe: – AUC: –	Median HU attenuation shift rather than mean yields better prediction accuracy and thus is preferable. It correlates well with clinical outcome in mRCC patients. A shift of median <–44 HU indicates a partial response while a shift of median >–41 HU indicates progression
Haider et al. [46]	To highlight potential radiomic predictors of progression-free survival (PFS) and overall survival (OS) that could be extracted from CECT images in RCC patients undergoing treatment with sunitinib (N = 40)	Shape: 1 – size change 1st-Order Statistics: 5 – MPP – STD – skewness – kurtosis – nSTD 2nd-Order Statistics: 1 – entropy	2D ROI CPHR (statistical analysis only)	Acc: – Sen: – Spe: – AUC: –	nSTD extracted from CECT before and after sunitinib treatment is positively correlated with both OS and PFS, while entropy and % size change are predictors of OS in RCC patients
Mains et al. [47]	To identify radiomic functional markers derived from CECT to act as potential predictors of OS and PFS in mRCC patients (N = 69)	Functional: 7 – BV_deconv_ – BF_deconv_ – SPV_deconv_ – BV_max_ – SPV_deconv_ – BV_patlak_ – PS	2D ROI MLEMD KM, LR, and Spearman ρ (statistical analysis only)	Acc: – Sen: – Spe: – AUC: –	Medians and modes of BV_deconv_, BV_patlak_, and BF_deconv_ are statistically significant (*p* < 0.05) and provide the strongest correlation with clinical outcome (PFS and OS)
Reynolds et al. [51]	To investigate the ability of radiomic markers extracted from DW-MRI (N = 12) and DCE-MRI (N = 10) as potential predictors of early treatment responses in RCC patients after stereotactic ablative body radiotherapy (SABR)	Shape: – tumor volume 1st-Order Statistics (DW-MRI): – mean ADC – median ADC – kurtosis – skewness Functional DCE-MRI: – mean T_onset_ – mean IRE – mean MaxE – mean T_wout_ – mean IRW – mean K_trans_ – % wout – % plateau – % presistent – % noenhance – iAUCAC60	3D ROI Spearman ρ (statistical analysis only)	Acc: – Sen: – Spe: – AUC: –	Statistically significant correlations between the change in percentage washout, change in mean IRE, and mean Ktrans, and the change in tumour volume (*p* < 0.05). Changes in ADC kurtosis showed statistically significant positive correlations with the percentage tumour volume change (*p* < 0.05)
Khodabakhshi et al. [48]	To explore the potential radiomic markers extracted from Phase 2 CECT and clinical biomarkers for the prediction of OS in RCC patients after partial or radical nephrectomy (N = 210)	Total: 225 Shape: 29 1st-Order Statistics: 50 2nd-Order Statistics: 136 – GLCM: – – GLRLM: – – GLSZM: – – GLDZM: – – NGTDM: – – NGLDM: –	2D ROI CPHR, AFT, bootstrapping, and KM (statistical analysis only)	Acc: – Sen: – Spe: – AUC: –	Besides tumor heterogeneity, grade, and stage as clinical indicators for OS, flatness, area density, and median are the most significant radiomic-based predictors (*p* < 0.05) of OS
Zhang et al. [49]	To investigate the prediction potentials of radiomics-based markers extracted from CECT images and clinical markers that are linked to progression-free survival (PFS) after partial or radical nephrectomy in ccRCC patients (N = 175)	Total: 6 Shape: 4 – Phase 1: 1 – Phase 2: 1 – Phase 3: 1 – Phase 4: 1 2nd-Order Statistics: 2 – Phase 2: 1 (GLSZM), 1 (NGTDM)	3D ROI LASSO, CPHR 29% validation	Acc: 0.70 Sen: 0.58 Spe: 0.74 AUC: 0.71	Radiomic-based markers extracted from CECT, especially Phase 2, demonstrated better prediction performance of PFS in ccRCC patients when combined with clinical markers (age, stage, and KPS score)

Notes: OS, overall survival; mRCC, metastatic renal cell carcinoma; KM, Kaplan–Meier; PFS, progression-free survival; SLR, simple linear regression; CPHR, Cox proportional hazards regression; HU, Hounsfield unit; VEGFR, vascular endothelial growth factor receptor; TKI, tyrosine kinase inhibitors; WRST, Wilcoxon signed-rank; nSTD, size-normalized standard; BV_deconv_, blood volume using deconvolution; BF_deconv_, blood flow using deconvolution; SPV_deconv_, standardize perfusion values using deconvolution; BV_max_, blood volume using maximum slope; SPV_deconv_, standardize perfusion values maximum slope; BV_patlak_, blood volume using the Patlak model; PS, permeability surface area product using the Patlak model; MLEMD, maximum likelihood expectation maximization deconvolution; *ρ*, Spearman’s rank correlation coefficient; T_onset_, time of onset of the contrast agent; IRE, initial rate of enhancement; MaxE, maximum enhancement; T_wout_, time of washout of the contrast agent; IRW, initial rate of washout; K_trans_, volume transfer constant between blood plasma and the extravascular extracellular space; iAUCAC60, initial area under the contrast agent concentration curve for the first 60 s postinjection; AFT, accelerated failure time; KPS, Karnofsky performance status.

## Abbreviations

The following summarizes the list of abbreviations that are used in this survey:

RC	Renal Cancer
RCC	Renal Cell Carcinoma
ccRCC	Clear-Cell RCC
nccRCC	Non-Clear-Cell RCC
paRCC	Papillary RCC
ChrRCC	Chromophobe RCC
AMLwvf	Angiomyolipoma without visible fat
ONC	Oncocytoma
CECT	Contrast-Enhanced Computed Tomography
CEMRI	Contrast-Enhanced Magnetic Resonance Imaging
DW-MRI	Diffusion-Weighted MRI
ADC	Apparent Diffusion Coefficient
AI	Artificial Intelligence
ML	Machine Learning
DL	Deep Learning
CAD	Computer-Aided Diagnosis
CAP	Computer-Aided Prediction
ROI	Region of Interest
AUC	Area Under the Curve
OS	Overall Survival
PFS	Progression-Free Survival
ANNs	Artificial Neural Networks
LR	Logistic Regression
RF	Random Forests
SVM	Support Vector Machine
CNN	Convolutional Neural Network
ROC	Receiver Operating Characteristics
MPP	Mean of Positive Pixels
SW-MRI	Susceptibility-Weighted MRI
GLCM	Gray-Level Co-occurrence Matrix
GLRLM	Gray-Level Run-Length Matrix
GLSZM	Gray-Level Size-Zone Matrix
NGTDM	Neighboring Gray-Tone Difference Matrix
GLDZM	Gray-Level Distance Zone Matrix
NGLDM	Neighboring Gray-Level Dependence Matrix
mRCC	Metastatic RCC
KM	Kaplan–Meier
PET	Positron Emission Tomography
SUV	Standard Uptake value
SABR	Stereotactic Ablative Body Radiotherapy
IRE	Initial Rate of Enhancement
MaxE	Maximum Enhancement
IRW	Initial Rate of Washout
iAUCAC60	Initial Area Under Contrast Agent Concentration Curve for 60 s postinjection
HUs	Hounsfield Units
VEGFR	Vascular Endothelial Growth Factor Receptor
LassoCV	Least Absolute Shrinkage and Selection Operator Cross-Validation
KPS	Karnofsky Performance Status
